# Dietary Supplementation with Microalgae (*Schizochytrium* sp.) Improves the Antioxidant Status, Fatty Acids Profiles and Volatile Compounds of Beef

**DOI:** 10.3390/ani11123517

**Published:** 2021-12-10

**Authors:** Chenchen Xu, Shou Zhang, Baozhong Sun, Peng Xie, Xiaochang Liu, Lan Chang, Fushan Lu, Songshan Zhang

**Affiliations:** 1 Institute of Animal Sciences, Chinese Academy of Agricultural Sciences, Beijing 100193, China; xuchenchen@caas.cn (C.X.); sunbaozhong@caas.cn (B.S.); xiepeng@caas.cn (P.X.); liuxiaochang@caas.cn (X.L.); 2 China Meat Research Center, Beijing Academy of Food Sciences, Beijing 100068, China; 3 College of Agriculture and Animal Husbandry, Qinghai University, Xining 810016, China; 1987990012@qhu.edu.cn (S.Z.); 1987990008@qhu.edu.cn (L.C.); 1993990012@qhu.edu.cn (F.L.)

**Keywords:** microalgae, docosahexaenoic acid, antioxidant status, n-3 polyunsaturated fatty acids, volatile compounds

## Abstract

**Simple Summary:**

This study evaluated the effects of *Schizochytrium* sp., as a promising feed additive, on the antioxidant enzyme activity, physicochemical quality, fatty acid composition and volatile compounds of beef. The results revealed that *Schizochytrium* could improve the antioxidant capacity of beef and increase the nutritional value, which was related to the abundant docosahexaenoic acid (DHA) found in *Schizochytrium*. Our current research results provide guidance for the development of environmentally friendly diet additives.

**Abstract:**

The purpose of this study was to evaluate the effects of dietary supplementation with microalgae (*Schizochytrium* sp.) containing docosahexaenoic acid (DHA) on the antioxidant enzyme activity, physicochemical quality, fatty acid composition and volatile compounds of beef meat. Eighteen male Qaidamford cattle were randomly allocated into three treatments (*n* = 6): no micro-algae supplementation (Control group, C), 100 g microalgae supplementation per bull per day (FD1), and 200 g microalgae supplementation per bull per day (FD2), and fed for 49 days before slaughter. The results showed that, compared with the C group, the addition of DHA-rich microalgae to the diet could significantly increase the total antioxidant capacity (T-AOC) in meat. In the FD2 group, it was found that the concentration of glutathione peroxidase (GSH-Px) was significantly higher than that of the control group (*p* < 0.05). DHA-rich microalgae supplementation increased polyunsaturated fatty acid (PUFA), eicosapentaenoic acid (EPA; C20:5 n-6), DHA, EPA + DHA, and n-3 PUFA and reduced n-6:n-3 fatty acid ratio. Twenty-four volatile compounds identified in beef were mainly aldehydes, alcohols and ketones from the fingerprints. The contents of short-chain fatty aldehydes, 1-octen-3-ol and 2-pentylfuran, were higher in the FD2 group than in the other two groups. The microalgae diet improved the sensory attribute score of beef. The results demonstrated that dietary supplementation of DHA-rich microalgae improved the antioxidant status, increased the deposition of DHA and enhanced the characteristic flavor of beef.

## 1. Introduction

The beef industry has become one of the fastest-growing industries in China’s animal husbandry [1]. Beef contributes high-quality protein to consumers. However, due to the biohydrogenation of n-3 polyunsaturated fatty acids (n-3 PUFA) in the ruminant rumen [2], the n-3 PUFA in beef is insufficient to meet the needs of human health [3]. It is well known that more n-3 PUFA, eicosapentaenoic acid (EPA) and docosahexaenoic acid (DHA) ingested in the diet, has health benefits for the control of human cardiovascular and cerebrovascular diseases [4]. The n-3 PUFA is an “essential” nutrient for the human body because humans cannot synthesize them [5]. Although α-linolenic acid (ALA) could be converted into EPA and DHA in the human body, it seems that the degree of conversion is limited, and the efficiency is extremely low [6,7]. Thus, it is an effective strategy to increase the concentration of DHA and EPA in beef by feeding the cattle n-3 PUFA-enriched fish oil or microalgae. 

Researchers found that after animals were fed fish oil, the oxidative stability of the meat was reduced and was accompanied by off-odors, which was caused by the oxidation of lipids in the meat during shelf life [8,9,10]. Another alternative approach is to add marine microalgae into animal diets. The advantage of this approach would be that microalgae may be photosynthetically autotrophic and grow fast [11,12]. They are used as a source of animal feed [13], thereby solving the problem of sustainability of supply [14]. In addition to the high content of DHA, microalgae also contain antioxidants such as β-carotenoids, vitamin E and vitamin A [15], which can enhance the immune response [16], antioxidant activity [17] and antibacterial effect [18] of animals, and ensure that animals remain healthy. *Schizochytrium* is a kind of thraustochytrid microalgae, and is the source of DHA in the marine food chain [19]. The addition of *Schizochytrium* in animal diets has been proven to improve the quality of meat and increase the content of n-3 PUFA [20,21,22,23]. Nevertheless, information on the relationship between microalgae and volatile compounds has not been reported. 

We hypothesized that the difference in antioxidant status and volatile compounds in beef after slaughter could be explained by the level of *Schizochytrium* addition. Therefore, the purpose of this study is to determine the influence of a dietary DHA-rich microalgae concentration through the determination of antioxidant capacity, physical indicators, fatty acid composition and volatile compounds.

## 2. Materials and Methods

### 2.1. Animals, Experimental Design and Diets

A total of 18 male Qaidamford cattle (24 months old, weighing 345.42 ± 12.49 kg) were randomly allocated to three experimental groups with 6 bulls of each, and assigned to individual pens (3 × 2.5 m). The experiment lasted for 49 days. This research was conducted in Qinghai Jinsui Animal Husbandry Co., Ltd. (Haixi Mongolian and Tibetan Autonomous Prefecture, Delingha City, China). All animals were fed twice daily at 08:00 h and 17:00 h. Water was provided freely. The experimental groups were as follows: (1) C (control group, a basal diet without microalgae powder supplementation), (2) FD1 (100 g microalgae powder per bull per day of basal diet) and (3) FD2 (200 g microalgae powder per bull per day of basal diet). The bulls first received the concentrate mixed with the *Schizochytrium* sp. powder and then ingested the dietary roughage mixed with oat hay and alfalfa hay. The ratio of forage to concentrate was 60:40. The ingredient and chemical composition of the basal diets are presented in Table 1. 

The crude protein, calcium and phosphorus contents of the experimental diet were analyzed following the Association of Official Analytical Chemists’ (AOAC) methods [24]. The concentrations of neutral detergent fiber and acid detergent fiber were calculated as described by Van Soest et al. [25]. The fatty acid (FA) profiles of the experimental diet were determined at the Ministry of Agriculture Feed Industry Center (College of Animal Science and Technology, China Agricultural University, Beijing, China), and analyzed by gas chromatography (Agilent 6890 Series, Agilent Technologies, Avondale, Palo Alto, CA, USA) equipped with a capillary column (CP-Sil 88 column, 0.25 mm × 50 m) according to the procedure described by Li et al. [26]. The FA composition of the *Schizochytrium* sp. used in the diets is shown in Table 2. The commercial *Schizochytrium* sp. powder (21.0% protein, 41.2% fat, 2.8% ash, and 2.3% moisture) was purchased from Xi’an Xiaocao Biotechnology Co., Ltd. (Xi’an, China).

### 2.2. Sample Collection

All animals were weighed (393.37 ± 23.53 kg for the C group, 408.50 ± 24.72 kg for the FD1 group, and 403.25 ± 15.94 kg for the FD2 group) and transported to commercial slaughterhouses for slaughter to facilitate sample collection and measurement after the end of the feeding trial. In order to better simulate the quality of beef consumed by residents in the Qinghai-Tibet Plateau, we chose 48 h after slaughter as the sampling time. After chilling for 48 h at 4 °C, the *M. longissimus lumborum* (LL) muscle sample was taken from the left side of each carcass and transported to the laboratory with a portable cooler. All beef samples were trimmed with visible fat and connective tissue before vacuum packaging and stored at −80 °C for further analysis within two weeks. 

### 2.3. Analysis of Antioxidant Enzymes Activity and Lipid Oxidation

All samples were thawed at 4 °C for 24 h before analysis. One gram of meat was mixed with cold phosphate-buffered saline (0.06 mol/L, pH 7.4) at a ratio of 1:10 (weight/volume, *w*/*v*) and then homogenized with Ultra Turrax T25 (IKA, Braun, Kronberg, Germany) homogenizer. After centrifugation at 4000 rpm and 4 °C for 10 min, the supernatant was collected to determine the protein content and antioxidant enzyme activity. Protein concentration, total antioxidant capacity (T-AOC), superoxide dismutase (SOD), glutathione peroxidase (GSH-Px) levels and thiobarbituric acid reactive substances (TBARS) level were measured using commercial assay kits (Nanjing Jiancheng Bioengineering Institute, Nanjing, China) by a spectrophotometer (Spectral Instrument Co. Ltd., Shanghai, China). The results of T-AOC, SOD and GSH-Px were expressed in U/mg protein, and the absorbance was measured at 520 nm, 550 nm and 412 nm, respectively. Lipid oxidation was evaluated using the TBARS value. The absorbance was measured at 532 nm. Results were expressed as mg malonaldehyde (MDA)/kg meat.

### 2.4. Meat Physicochemical Quality Characteristics Analysis 

#### 2.4.1. pH and Color Measurement

The pH values were measured with a Testo 205 pH meter (Lenzkirch, Germany) calibrated with pH 4 and 7 standard buffer solutions. The average value obtained by inserting electrodes into three different points of each sample was used for statistical analysis.

The thawed LL muscle sample was bloomed for 45 min before measurement. The surface color of meat was measured with a portable CR-400 Colorimeter (Minolta Inc., Osaka, Japan), using an illuminant D65 and a 2-degree standard observer. Color parameters L* (lightness), a* (redness) and b* (yellowness) were measured at different positions on the surface of each sample with triplicate measurements, and the average values were calculated. Chroma (C*) and hue angle (H*) were evaluated by the following equations:

C* = (a*^2^ + b*^2^)^1/2^, (1)

H* = arctan (b*/a*) × (180/π) (2)

#### 2.4.2. Drip Loss and Cooking Loss Determination

Drip loss. Drip loss was estimated according to the protocol followed by Honikel [27]. Approximately 60 g of each meat sample was weighed (W1) and then suspended in an inflated polyethylene bag at 4 °C without any contact with the bag. After 24 h, the sample was taken out of the bag, gently wiped off the residual surface drip, and then weighed (W2). Drip loss was calculated as the percentage ratio of the initial weight using the following formula:

Drip loss = (W1 − W2)/W1 × 100%,(3)

Cooking Loss. Cooking loss was determined as suggested by Fabre et al. [28]. Briefly, the thawed sample was weighed (M1) and placed in a polyethylene bag, and then cooked under boiling water (98 ± 1 °C) until the internal temperature reached 71 °C. The sample was taken out and weighed after cooling (M2). The cooking loss was expressed as a percentage of the initial sample weight, according to the following formula:

Cooking loss = (M1 − M2)/W1 × 100%,(4)

#### 2.4.3. Proximate Composition Analysis

Approximately 50 g of the minced meat sample was measured according to the method of the AOAC [24], in which protein was analyzed by a Kjeldahl K9840 analyzer (Hanon Instrument Co. Ltd., Nanjing, China), and fat was determined by an Ankom XT15 analyzer (Ankom Technology, Macedon, NY, USA). All measurements were taken in triplicates.

### 2.5. Fatty Acids Analysis

The fatty acids composition analyses were carried out according to Ponnampalam et al. [29]. In brief, the beef samples collected from each group were sliced and freeze-dried in a freeze dryer (Ningbo Xinzhi Instruments, Inc., Ningbo, China) for 72 h. Freeze-dried samples (0.5 g) were ground, and then 1 mL internal standard (C11:0, Sigma Aldrich Pty Ltd., St. Louis, MI, USA) was added to muscle samples. The contents were hydrolyzed with 0.7 mL of 10 mol/L potassium hydroxide aqueous solution and 5.3 mL of methanol to form free fatty acids. Then, the contents were incubated in a water bath at 55 °C for 1.5 h by vortex mixing and mixed with 0.58 mL of 24 N sulfuric acid (H_2_SO_4_) in water and cooled to room temperature. After 1.5 h incubation, the mixture was cooled and then thoroughly mixed with 3 mL of hexane solvent and centrifuged. Approximately 1 mL supernatant (fatty acid methyl ester, FAME) was transferred into an injection vial for analysis. The FAME was determined with the gas chromatograph (GC-6890 N, Agilent Technologies, Wilmington, NC, USA), equipped with a detector (flame ionization), and separate SP-2560 type column (capillary) (60 m × 2.5 cm × 0.25 μm, Supelco Inc., Bellefonte, PA, USA). The sample split ratio was 30:1. Gas chromatographic conditions were set according to Aldai et al. [30]. The ratio of retention time to the FAME standard mixture (FAME 37 component, Sigma-Aldrich Co., St. Louis, MI, USA) was used to identify the fatty acids.

### 2.6. Volatile Compounds Analysis

Beef samples were collected in triplicate for each group of volatile compounds. The volatile compounds were determined using a Flavorspec Gas Chromatograph-Ion Mobility Spectrometer (GC-IMS) system (GAS GmbH, Dortmund, Germany) fitted with a SE-54 capillary column (15 m × 0.53 mm), and conducted with modifications according to the methods of Xu et al. [31]. Briefly, without any sample pre-treatment, two grams of fresh meat samples were weighed and placed in a 20 mL headspace vial and incubated at 60 °C for 20 min. Then, 500 L of headspace was automatically injected into the heated syringe with a syringe at 85 °C. High-purity nitrogen was used as the carrier gas with a flow rate of 150 mL/min. The carrier gas flow rate program was set to 2 mL/min for 2 min; 10 mL/min for 10 min, 100 mL/min for 20 min, and 150 mL/min for 25 min. LAV software (version 2.2.1—G.A.S., Dortmund, Company) was used to create an area set integrating retention time (Rt) and drift time (Dt) of all markers spots and to obtain the individual signal from the topographic plot.

### 2.7. Sensory Analysis

Eight trained sensory panel members were selected according to the Chinese standard GB/T 22210-2008 (criterion for the sensory evaluation of meat and meat products). The samples were baked on a household grill at 185 °C and removed when the core temperature reached 70 °C. After cooking, each sample was cut into 1.5 cm cubes, placed on a paper plate with a three-digit number and then presented to the panelists. During testing, panelists were seated in a separate compartment in the sensory room. Before proceeding to the next round of evaluation, panelists were instructed to rinse their mouths with water to clear the taste between the samples. All sensory analyses were repeated three times. The sensory attribute score of each sample was divided into 8 levels: initial juiciness (1 = extremely dry, 8 = extremely juicy); sustained juiciness (1 = extremely dry, 8 = extremely juicy); flavor intensity (1 = extremely bland, 8 = extremely intense); off-flavor intensity (1 = no off-flavor, 8 = extremely intense off-flavor); initial tenderness (0 = extremely tough, 8 = extremely tender); sustained tenderness (0 = extremely tough, 8 = extremely tender); residue (0 = none, 8 = abundant).

### 2.8. Statistical Analysis

All the experiments were performed in triplicate. The collected data were analyzed using one-way analysis of variance (ANOVA) by the SAS 9.2 program (SAS Institute, Cary, NC, USA). The Shapiro–Wilk test and Levene test were used to evaluate the normality of the data distribution and the homogeneity of variance. The Duncan test was used to compare mean values. Significant differences were declared at *p* < 0.05.

## 3. Results

### 3.1. Antioxidant Enzymes Activity and Lipid Oxidation 

**Figure 1:**
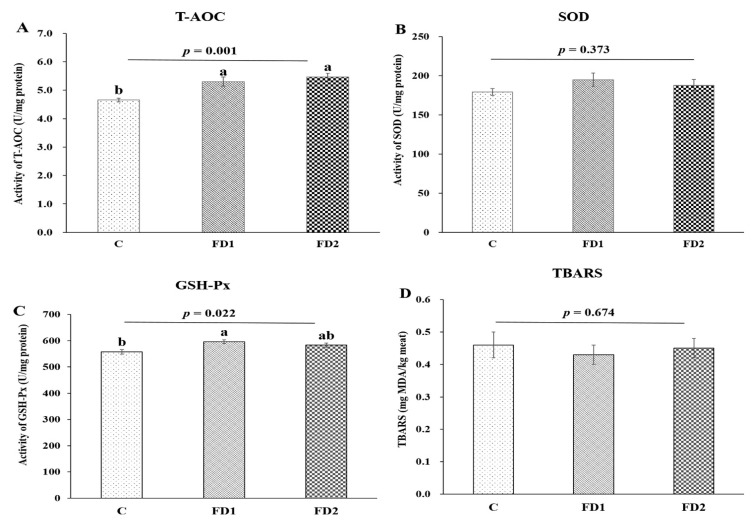
The influence of dietary DHA-rich microalgae on the activities of (**A**) T-AOC, (**B**) SOD, (**C**) GSH-Px and (**D**) TBARS of beef. C = control group; FD1 = 100 g microalgae powder per bull per day of basal diet; FD2 = 200 g microalgae powder per bull per day of basal diet. Different letters indicate significant differences due to DHA-rich microalgae supplementation levels (*p* < 0.05).

Effects of dietary DHA-rich microalgae on the antioxidant enzyme activity of beef are shown in Figure 1. Compared with the control group, the activities of T-AOC in the FD1 group and FD2 group and GSH-Px in the FD1 group significantly increased (*p* < 0.05). No significant difference was observed in T-AOC and GSH-Px activities between the FD1 group and the FD2 group (*p* > 0.05). There was no significant difference in SOD activity and TBARS in all treatment groups (*p* > 0.05).

### 3.2. Meat Physicochemical Quality

The effects of dietary DHA-rich microalgae on physicochemical meat quality are presented in Table 3. There were no differences (*p* > 0.05) in color parameters (L*, a*, b*, C* and H*), pH, drip loss, cooking loss and protein among the three treatments. The fat content in the FD1 and FD2 groups was significantly higher than that in the control group (*p* < 0.05).

### 3.3. Fatty Acid Composition

The influences of dietary DHA-rich microalgae on fatty acids composition in the LL muscle of beef are given in Table 4. It could be seen that dietary DHA-rich microalgae treatments had no effects on monounsaturated fatty acids (MUFA) and n-6 PUFA (*p* > 0.05). Saturated fatty acids (SFA) in the FD1 and FD2 groups was greater (*p* < 0.05) than that in the control group. The cattle supplemented with DHA-rich microalgae had higher SFA (*p* = 0.007), PUFA (*p* = 0.001), n-3 PUFA (*p* < 0.001) and EPA + DHA (*p* < 0.001), and a lower (*p* < 0.001) n-6/n-3 ratio. Moreover, the concentrations of EPA, DHA, EPA + DHA and total n-3 PUFA increased significantly with increasing DHA-rich microalgae (*p* < 0.001). The relative proportion of EPA and DHA were 4–6 times and 8.5–11.4 times higher (*p* < 0.001) for FD1 and FD2 groups compared to the control group.

Diet also influenced individual fatty acid contents, whereas C16:0 and C18:3 n-3 (ALA) in the FD1 and FD2 groups was higher (*p* < 0.05) than that in the control group. DHA-rich microalgae increased the concentrations of C16:0 and SFA in the meat, which may be related to the higher contents of C16:0 and SFA contained in microalgae powder, which were difficult to be oxidized and easy to deposit [15]. The concentrations of C20:0 and C16:1 in the FD2 group were greater (*p* < 0.05) than that in the control group, but there was no difference (*p* > 0.05) from that in the FD1 group.

### 3.4. Volatile Compounds 

**Figure 2:**
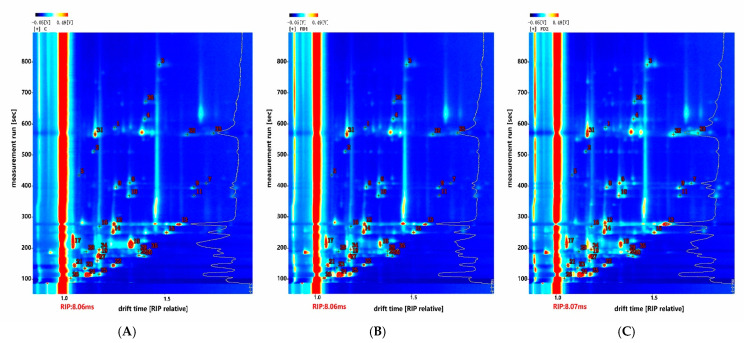
Ion migration spectra of beef meat under different treatments. RIP, reactive ion peak. (**A**) C, control group; (**B**) FD1, 100 g microalgae powder per bull per day of basal diet; (**C**) FD2, 200 g microalgae powder per bull per day of basal diet.

As shown in Figure 2, the abscissa was used to represent the drift time, and the ordinate was used to represent the retention time. The drift time and retention time were compared to indicate the volatile compounds in the beef sample. Each spot represented a kind of volatile compound. The identified compounds are presented in Table 5. A total of 36 peaks and 24 volatile components were identified from the beef samples of the three groups, including 9 alcohols, 8 aldehydes, 6 ketones and 1 furan. Among them, 11 were dimers of the compounds, and 1 was a trimer of the detected compounds. 

**Figure 3:**
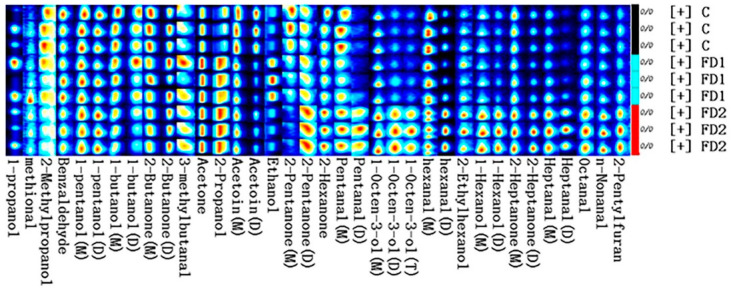
Gallery plots of volatile compounds of beef treated with different levels of DHA-rich microalgae. All signal strengths are expressed in color. The redder the spot indicates the greater the amount of volatile compounds detected. C, control group; FD1, 100 g microalgae powder per bull per day of basal diet; FD2, 200 g microalgae powder per bull per day of basal diet.

To further study the flavor difference between beef samples after adding microalgae, the fingerprints obtained are shown in Figure 3. The fingerprint was formed based on the peak signal. Each row represented the sample, and each column represented the identified volatile compounds. In the fingerprint, the more pronounced color indicated, the higher the content of the flavor substance identified. The contents of 2-propanol and acetone in the FD1 and FD2 groups were much higher than that in the control group. In the FD2 group, it was found that the concentrations of many compounds in beef greatly increased, such as 1-hexanol, 1-octen-3-ol, 2-ethylhexanol, n-nonanal, octanal, heptanal, hexanal (dimer), pentanal (dimer), 2-heptanone and 2-pentylfuran. However, the concentrations of acetoin, 2-pentanone (monomer) and 2-butanone (monomer) were the highest in the control group. 

### 3.5. Sensory Evaluation

The mean scores for sensory evaluation of beef are presented in Table 6. Compared with the beef samples of the control group, the beef samples of the FD1 and FD2 groups showed higher (*p* < 0.05) scores of initial juiciness, sustained juiciness, flavor intensity, initial tenderness and sustained tenderness, and lower (*p* < 0.05) scores of residue. There was no observed effect of diet (FD1 or FD2) on off-flavor intensity (*p* > 0.05). However, the sensory scores between the FD1 and FD2 groups did not show a significant difference (*p* > 0.05).

## 4. Discussion

It is known that antioxidant capacity is an important factor in maintaining animal health [32]. As a part of the endogenous antioxidant defense system, antioxidant enzymes played a key role, which could protect cells from oxidative damage by free radicals [33]. A previous study showed that *Schizochytrium* contained some antioxidants such as β-carotene and vitamin E [15]. SOD directly reacts with free radicals to remove hydrogen peroxide [34], while GSH-Px catalyzes the decomposition of hydrogen peroxide [35]. In this study, compared with the control group, the addition of DHA-rich microalgae increased the T-AOC of beef meat, which indicated that DHA-rich microalgae could improve the antioxidant status of beef. It was found that only the GSH-Px activity in the FD1 group increased in the current study, which suggested that the antioxidants in the microalgae at an appropriate dose might contribute to the stability of the meat after slaughter. Adding DHA-rich microalgae to the diet did not affect the SOD activity of beef. This finding is consistent with La et al. [36], who reported that supplementing the diet with *Schizochytrium* did not affect the SOD activity in the blood of calves. The content of TBARS is considered to be one of the most accurate indicators for evaluating lipid peroxidation [37]. Since long-chain PUFA was the main target of reactive oxygen species, it was determined that the risk of lipid oxidation of beef was increased [38]. Our results showed that the addition of DHA-rich microalgae in the diet did not affect TBARS. This result could be explained that the increase in antioxidant capacity and endogenous antioxidant enzyme activity (GSH-Px) scavenged free radicals to protect PUFA, resulting in no lipid peroxidation, although the concentration of PUFA of beef meat increased after adding microalgae to the diet.

Meat color is an important indicator for evaluating meat quality, which determines the consumers’ desire to buy [39]. In the present research, no changes in beef meat color between groups were observed, which is consistent with previous studies. They reported that adding DHA-rich *Schizochytrium* did not affect the animal meat color [40,41,42]. The lack of differences in the ultimate pH between the treatments indicates that the muscle glycogen levels of the beef in the three treatment groups are similar. Drip loss and cooking loss were used to evaluate the water holding capacity of the meat. It should be noted that no difference in water retention was observed between the three treatments. The amount of intramuscular fat is related to eating qualities such as juiciness, tenderness and flavor [43]. Urrutia et al. [44] reported that dietary microalgae changed adipose tissue development and cell structure, which may be an important factor in causing fat deposition in this study. Generally, the strategy of using microalgae as a diet does not affect meat quality parameters [45], but it could improve the fatty acid composition of the meat. This deserves more attention and further research.

With the increase of DHA-rich microalgae supplemented in the diet, the contents of EPA, DHA, EPA + DHA and n-3 PUFA showed a linear increase. As expected, marine resources exhibited the ability to accumulate DHA and EPA contents in meat [46]. There was evidence that adding algae to the diet was easier to obtain DHA deposition than through the desaturation and prolongation pathway of α-Linolenic acid (C18:3 n-3, ALA) [47]. Based on the Australian nutrient reference standard, if the EPA + DHA in red meat exceeds 60 mg/135 g meat, it could be considered as a good source of n-3 [44]. The European standard for a good source of n-3 recognized EPA + DHA as 40 mg and 80 mg/100 g of meat [48]. In this experiment, the content of EPA + DHA in beef (60.30 mg/100 g fresh meat and 80.19 mg/100 g fresh meat) was sufficient to meet this standard, which meant that the beef in this study could provide enough EPA and DHA for the healthy human diet. The balance of the ratio of n-6 and n-3 PUFA is a key factor for maintaining human health [49]. Burghardt et al. reported that the n-6/n-3 PUFA ratio in the human diet should not exceed 4 [50]. In this study, the ideal ratio of n-6/n-3 in beef meat in the FD1 and FD2 groups illustrated that this beef meat could help reduce the risk of cardiovascular and cerebrovascular diseases and certain cancers after being consumed by humans [51]. The changes in fatty acid composition observed in this study may be attributed to the differences in fatty acid metabolism and deposition of beef with different dietary treatments [52]. In addition, in the absence of C18:3 n-6 in the diet, the C18:3 n-6 detected in beef meat may be attributed to the conversion of C18:2 n-6 through elongation and desaturation from certain diets in this experiment [53]. 

Volatile alcohols, aldehydes and ketones belong to lipid-derived compounds [54]. They are the simplest product of lipid degradation and are formed by the modification of fatty acids after removal from the glycerol backbone [55]. In this experiment, short-chain fatty aldehydes content was found to be the highest in the FD2 group, such as n-nonanal, octanal, heptanal, hexanal and pentanal, and hexanal was the highest amount of them. Most aldehydes are mainly derived from oleic acid (C18:1 n-9) and linoleic acid (C18:2 n-6), but nonanal was only produced from oleic acid [56]. The results clearly illustrate that the high correlation between the flavor compound and fatty acid composition, and the decisive role of lipids in the formation of basic meat flavor. Previous studies showed that aldehydes were the main volatile compounds in beef meat [57,58]. Volatile compounds were mainly affected by diet because the fatty acid composition of meat was changed by diet [59]. Therefore, this also explained the higher aldehyde content in the FD2 group. Hexanal was the main volatile compound in beef meat and directly related to lipid auto-oxidation [60]. The alcohols observed in the experiment were secondary products of aldehydes. The FD1 group showed a higher amount of ethanol. It is interesting that due to the higher alcohol threshold, the contribution to the undesirable flavor of the meat was less [61]. However, the highest content of 1-octen-3-ol in the FD2 group could indicate a mushroom flavor, which could be perceived as a mushroom-related flavor. The highest value of 2-pentylfuran (beany and grassy flavor) was observed in the FD2 group, which could be formed by lipid oxidation and degradation. It was an oxygen-containing heterocyclic compound with a low threshold, resulting in a greater contribution to the overall flavor of the meat [62]. Prior work has shown that ketones were derived from the β-oxidation of free fatty acids increased with fat content in meat [63]. Acetoin (buttery flavor), 2-butanone and 2-pentanone levels in the C group were significantly higher than the other two groups, suggesting that the fat content was reduced by DHA-rich microalgae. To our knowledge, there is no information on the effect of an algae diet on volatile beef compounds. In the current study, high levels of volatile compounds were associated with high levels of DHA in the meat of algae-fed animals. 

Most consumers believe that higher fat is related to greater eating satisfaction [64]. This study found that consumers’ liking of tenderness, juiciness and flavor were affected by diet. The aldehydes produced by the Strecker degradation of the Maillard reaction were generally considered to be positive flavors associated with cooking [65]. This is consistent with the results of volatile substances in this study. There was no difference in the off-flavor scores of the three groups, which might be related to the TBARS value in the meat (less than 0.5 mg MDA/kg meat) [66]. This study proved that adding DHA-rich microalgae to the diet could reduce the development of off-flavor by increasing oxidative stability.

## 5. Conclusions

In conclusion, adding different levels of DHA-rich microalgae to the diet could increase the activity of certain antioxidant enzymes in beef. Our results demonstrate that DHA and EPA levels in meat were enriched by supplementing DHA-rich microalgae. In addition, the reduced ratio of n-6/n-3 was another potential health benefit associated with algae in *Schizochytrium* sp. At the same time, the supplementation of DHA-rich microalgae enhanced the volatile compounds in beef, especially in the high-dose FD2 group. It is necessary to conduct further research on the metabolites and oxidation mechanism of fatty acids in meat.

## Figures and Tables

**Table 1:** Ingredients and chemical compositions of experimental diets.

Ingredients (g/kg Fed Basis)		Chemical Composition (g/kg DM Basis) ^2^	
Oat Hay	200	Crude protein	122.0
Alfalfa hay	400	Neutral detergent fiber	315.5
Corn	216	Acid detergent fiber	169.0
Wheat bran	24	Calcium	5.2
Wheat distillers dried grains with solubles	20	Phosphorus	4.0
Soybean meal	48	Net energy/(MJ/kg) ^3^	5.3
Rapeseed meal	40		
Cottonseed meal	16		
Jujube powder	12		
Fatty Acid Calcium	4		
Sodium Chloride	4		
Mineral and vitamin premix ^1^	16		
Fatty Acids, mg/g Diet			
C14:0	0.13	C16:1	0.10
C15:0	0.02	C18:1 n-9	12.08
C16:0	9.19	C18:2 n-6	16.44
C17:0	0.05	C18:3 n-3 (ALA)	0.94
C18:0	1.03	C20:1	0.16
C20:0	0.17	C20:2 n-6	0.01
C21:0	0.02	C20:5 n-3 (EPA)	0.02
C22:0	0.10		

DM, dry matter; ALA, α-linolenic acid; EPA, eicosapentaenoic acid. ^1^ Vitamin and mineral premix supplied each kg of feeds with Vitamin A 4000 IU; Vitamin D3 300 IU; Vitamin E 45 IU; Cu 8 mg; Fe 48 mg; Mn 30 mg; Zn 25 mg; I 0.2 mg; Se 0.3 mg; Co 0.12 mg. ^2^ Analyzed value. ^3^ Calculated value.

**Table 2:** The fatty acid composition of *Schizochytrium* sp. used in diets.

Fatty Acid, mg/g Dried Powder	
C14:0	1.96
C15:0	5.07
C16:0	73.04
C17:0	4.33
C18:0	3.26
C20:0	0.84
C21:0	0.02
C22:0	0.53
C23:0	0.11
C16:1	0.56
C18:1 n-9	0.03
C20:1	0.06
C18:2 n-6	0.15
C20:2 n-6	0.24
C18:3 n-3 (ALA)	0.97
C20:3 n-6	0.82
C20:4 n-6	0.59
C20:5 n-3 (EPA)	1.90
C22:6 n-3 (DHA)	202.62

ALA, α-linolenic acid; EPA, eicosapentaenoic acid; DHA, docosahexaenoic acid.

**Table 3:** The influence of dietary DHA-rich microalgae on the physicochemical quality of beef meat.

Item	Treatments			SEM	*p*-Value
	C	FD1	FD2		
L*	30.07	29.52	29.73	1.72	0.66
a*	15.17	14.34	15.73	2.51	0.64
b*	6.37	6.72	6.94	1.67	0.84
C*	16.47	15.93	17.23	2.66	0.70
H*	22.51	25.60	23.61	5.17	0.59
pH	6.1	6.04	6.19	0.522	0.885
Drip loss (%)	1.97	1.78	1.56	0.44	0.294
Cooking loss (%)	0.29	0.31	0.26	0.04	0.126
Protein (g/100 g)	23.84	23.59	23.91	1.11	0.875
Fat (g/100 g)	1.35 ^b^	1.76 ^a^	1.82 ^a^	0.27	0.047

SEM, standard error of means. C, control group; FD1, 100 g microalgae powder per bull per day of basal diet; FD2, 200 g microalgae powder per bull per day of basal diet. ^a,b^ Means within a row with different superscripts differ (*p* < 0.05).

**Table 4:** The influence of dietary DHA-rich microalgae on fatty acid profiles of beef meat.

Item (mg/100 g Fresh Meat)	Treatments			SEM	*p*-Value
	C	FD1	FD2		
C14:0	17.64	23.00	24.99	8.11	0.297
C15:0	6.85	7.34	8.86	1.66	0.111
C16:0	248.47 ^b^	327.77 ^a^	336.94 ^a^	59.86	0.042
C17:0	14.39	19.11	19.06	4.64	0.164
C18:0	236.01	331.83	357.42	86.44	0.065
C20:0	2.86 ^b^	3.35 ^ab^	4.41 ^a^	0.97	0.041
C21:0	1.71	1.89	1.39	0.53	0.285
C22:0	0.08	0.07	0.07	0.01	0.752
C23:0	1.01	1.11	1.16	0.19	0.386
SFA	529.03 ^b^	715.48 ^a^	754.29 ^a^	111.74	0.007
C15:1	82.88	90.44	86.85	17.87	0.768
C16:1	20.08 ^b^	27.86 ^a,b^	36.46 ^a^	7.12	0.004
C18:1 n-9	213.31	222.08	286.45	91.73	0.347
C20:1	1.88	1.90	1.98	0.64	0.962
C22:1	1.60	1.63	2.02	0.45	0.230
MUFA	319.75	343.92	413.76	104.80	0.301
C18:2 n-6	165.56	176.09	160.43	35.64	0.744
C18:3 n-6	1.29	1.54	1.20	0.28	0.128
C18:3 n-3 (ALA)	5.31 ^b^	6.33 ^a^	6.82 ^a^	0.74	0.009
C20:2 n-6	5.09	5.02	5.68	1.26	0.614
C20:3 n-6	13.39	11.86	11.16	2.31	0.261
C20:4 n-6	73.76	74.48	85.72	9.91	0.096
C20:5 n-3 (EPA)	3.46 ^c^	12.34 ^b^	18.87 ^a^	2.44	<0.001
C22:6 n-3 (DHA)	7.33 ^c^	60.30 ^b^	80.19 ^a^	5.24	<0.001
PUFA	275.19 ^b^	351.51 ^a^	370.09 ^a^	36.86	0.001
n-6 PUFA	259.10	268.98	264.20	36.54	0.897
n-3 PUFA	16.10 ^c^	78.97 ^b^	105.89 ^a^	6.19	<0.001
EPA + DHA	10.79 ^c^	72.64 ^b^	99.06 ^a^	6.28	<0.001
n-6/n-3	16.15 ^a^	3.43 ^b^	2.52 ^b^	1.13	<0.001

SEM, standard error of means; SFA, saturated fatty acids; MUFA, monounsaturated fatty acids; PUFA, polyunsaturated fatty acids; DHA, docosahexaenoic acid; EPA, eicosapentaenoic acid; C, control group; FD1, 100 g microalgae powder per bull per day of basal diet; FD2, 200 g microalgae powder per bull per day of basal diet. Within a row, values with different superscript letters are significantly different. Different superscripts within a row differ significantly (*p* < 0.05). SFA = C14:0 + C15:0 + C16:0 + C17:0 + C18:0 + C20:0 + C21:0 + C22:0 + C23:0. MUFA = C15:1 + C16:1 + C18:1 n-9 + C20:1 + C22:1. PUFA = C18:2 n-6 + C18:3 n-6 + C20:2 n-6 + C20:3 n-6 + C20:4 n-6 + C18:3 n-3 + C20:5 n-3 + C22:6 n-3. n-6 PUFA = C18:2 n-6 + C18:3 n-6 + C20:2 n-6 + C20:3 n-6 + C20:4 n-6. n-3 PUFA = C18:3 n-3 + C20:5 n-3 + C22:6 n-3.

**Table 5:** The information on identified volatile compounds of beef meat (36 peaks for 24 compounds).

Number	Compound	CAS^#^	Formula	MW	RI	Rt [s]	Dt [ms]
1	2-Pentylfuran	C3777693	C9H14O	138.2	995.0	588.195	1.25804
2	Benzaldehyde	C100527	C7H6O	106.1	958.7	511.489	1.15827
3	n-Nonanal	C124196	C9H18O	142.2	1103.3	790.923	1.47531
4	Octanal	C124130	C8H16O	128.2	1011.5	616.193	1.40526
5	Methional	C3268493	C4H8OS	104.2	916.8	435.272	1.08542
6	Heptanal (Monomer)	C111717	C7H14O	114.2	900.6	409.009	1.33262
7	Heptanal (Dimer)	C111717	C7H14O	114.2	900.0	408.054	1.7041
8	2-heptanone (Monomer)	C110430	C7H14O	114.2	892.3	396.116	1.26633
9	2-Heptanone (Dimer)	C110430	C7H14O	114.2	891.9	395.639	1.64334
10	1-Hexanol (Monomer)	C111273	C6H14O	102.2	871.8	368.421	1.32986
11	1-Hexanol (Dimer)	C111273	C6H14O	102.2	872.1	368.898	1.64472
12	Hexanal (Monomer)	C66251	C6H12O	100.2	792.4	278.226	1.25507
13	Hexanal (Dimer)	C66251	C6H12O	100.2	791.8	277.602	1.57443
14	1-Pentanol (Monomer)	C71410	C5H12O	88.1	764.5	251.354	1.24767
15	1-Pentanol (Dimer)	C71410	C5H12O	88.1	762.4	249.479	1.51031
16	2-Hexanone	C591786	C6H12O	100.2	783.7	269.79	1.18602
17	Acetoin (Monomer)	C513860	C4H8O2	88.1	716.1	210.419	1.05532
18	Acetoin (Dimer)	C513860	C4H8O2	88.1	713.6	208.544	1.34015
19	1-Butanol (Monomer)	C71363	C4H10O	74.1	663.8	177.922	1.18356
20	1-Butanol (Dimer)	C71363	C4H10O	74.1	659.9	176.047	1.37591
21	2-Butanone (Monomer)	C78933	C4H8O	72.1	581.6	142.612	1.06395
22	2-Butanone (Dimer)	C78933	C4H8O	72.1	588.9	145.424	1.25137
23	1-Propanol	C71238	C3H8O	60.1	560.7	134.8	1.11204
24	Acetone	C67641	C3H6O	58.1	489.8	111.364	1.1256
25	2-Propanol	C67630	C3H8O	60.1	501.1	114.802	1.18232
26	Ethanol	C64175	C2H6O	46.1	458.3	102.303	1.04546
27	2-Methylpropanol	C78831	C4H10O	74.1	620.3	158.236	1.17492
28	2-Pentanone (Monomer)	C107879	C5H10O	86.1	685.3	188.546	1.12067
29	2-Pentanone (Dimer)	C107879	C5H10O	86.1	685.8	188.78	1.37521
30	3-Methylbutanal	C590863	C5H10O	86.1	652.1	172.421	1.40027
31	1-Octen-3-ol (Monomer)	C3391864	C8H16O	128.2	985.9	567.857	1.16379
32	1-Octen-3-ol (Dimer)	C3391864	C8H16O	128.2	984.8	565.584	1.61059
33	1-Octen-3-ol (Trimer)	C3391864	C8H16O	128.2	987.3	571.04	1.73824
34	Pentanal (Monomer)	C110623	C5H10O	86.1	695.9	195.421	1.18443
35	Pentanal (Dimer)	C110623	C5H10O	86.1	694.3	194.228	1.42808
36	2-Ethylhexanol	C104767	C8H18O	130.2	1043.5	672.122	1.40894

CAS^#^, the registration number of chemical substances; MW, molecular mass; RI, retention index; Rt, retention; Dt, drift time.

**Table 6:** Effect of dietary DHA-rich microalgae on sensory characteristics of beef.

Item	Treatments			SEM	*p*-Value
	C	FD1	FD2		
Initial juiciness	5.13 ^b^	6.19 ^a^	6.21 ^a^	0.56	0.006
Sustained juiciness	4.94 ^b^	5.94 ^a^	6.10 ^a^	0.63	0.012
Flavor intensity	5.48 ^b^	6.33 ^a^	6.44 ^a^	0.45	0.004
Off-flavor intensity	2.17	2.00	2.31	0.28	0.187
Initial tenderness	5.52 ^b^	6.33 ^a^	6.50 ^a^	0.60	0.029
Sustained tenderness	5.63 ^b^	6.35 ^a^	6.42 ^a^	0.56	0.049
Residue	3.68 ^a^	3.21 ^b^	3.10 ^b^	0.34	0.022

SEM, standard error of means. C, control group; FD1, 100 g microalgae powder per bull per day of basal diet; FD2, 200 g microalgae powder per bull per day of basal diet. ^a,b^ Means within a row with different superscripts differ (*p* < 0.05).

## Data Availability

Data are available from the corresponding author upon request.
